# The Prognostic Model Established by the Differential Expression Genes Based on CD8^+^ T Cells to Evaluate the Prognosis and the Response to Immunotherapy in Osteosarcoma

**DOI:** 10.1155/2023/6563609

**Published:** 2023-02-09

**Authors:** Yu Chen, Wei Yan, Hongqing Wang, Zhiliang Ou, Huihong Chen, Zhenhua Huang, Jinlian Yang, Biqiong Liu, Fanjie Ou, Huang Zhang

**Affiliations:** ^1^The Third Department of Surgery, Xianyou County General Hospital, Putian, Fujian, China; ^2^Xianyou County General Hospital, Putian, Fujian, China; ^3^The Second Orthopedic Rehabilitation Center, Beijing Rehabilitation Hospital, Capital Medical University, Beijing, China; ^4^The Department of Neurosurgery, Xianyou County General Hospital, Putian, Fujian, China; ^5^Pulmonary and Critical Care Medicine, Xianyou County General Hospital, Putian, Fujian, China; ^6^Department of Nephrology, Xianyou County General Hospital, Putian, Fujian, China

## Abstract

Osteosarcoma (OS) is a malignant tumor with an extremely poor prognosis, especially in progressive patients. Immunotherapy based on immune checkpoint inhibitors (ICIs) is considered to be a promising treatment option for OS. Due to tumor heterogeneity, only a minority of patients benefit from immunotherapy. Therefore, it is urgent to explore a model that can accurately assess the response of OS to immunotherapy. In this study, we obtained the single-cell RNA sequencing datasets of OS patients from public databases and defined 34 cell clusters by dimensional reduction and clustering analysis. PTPRC was applied to identify immune cell clusters and nonimmune cell clusters. Next, we performed clustering analysis on the immune cell clusters and obtained 25 immune cell subclusters. Immune cells were labeled with CD8A and CD8B to obtain CD8^+^ T cell clusters. Meanwhile, we extracted the differentially expressed genes (DEGs) of CD8^+^ T cell clusters and other immune cell clusters. Furthermore, we constructed a prognostic model (CD8-DEG model) based on the obtained DEGs of CD8^+^ T cells, and verified the excellent predictive ability of this model for the prognosis of OS. Moreover, we further investigated the value of the CD8-DEG model. The results indicated that the risk score of the CD8-DEG model was an independent risk factor for OS patients. Finally, we revealed that the risk score of the CD8-DEG model correlates with the immune profile of OS and can be used to evaluate the response of OS to immunotherapy. In conclusion, our study revealed the critical role of CD8 cells in OS. The risk score model based on CD8-DEGs can provide guidance for prognosis and immunotherapy of OS.

## 1. Introduction

Osteosarcoma (OS) is a rare and highly lethal malignancy, accounting for more than 50% of malignant primary bone tumors [1]. OS originates from primitive mesenchymal cells and occurs mostly in the metaphysis of long bones, including the distal femur and proximal tibia [2]. OS is also the second leading cause of tumor-related deaths in children and adolescents after lymphoma and brain tumors [3]. It is widely recognized that environmental factors and genetic mutations are high-risk factors for OS [4]. Given that tumors are regulated by complex gene networks, the pathogenesis of OS has not been fully elucidated. Surgery, chemotherapy, and radiotherapy are still the classic treatment options for OS currently [5]. For patients with local OS, traditional treatment regimens can achieve a 5-year survival rate of more than 70% [6]. However, for OS patients with recurrence and metastasis, the 5-year survival rate does not exceed 20% [7]. Therefore, it is extremely urgent to explore a new treatment method that can fundamentally improve the prognosis of OS.

An increasing number of studies have shown that the tumor immune microenvironment (TIM) play a vital role in the occurrence and development of tumors, including OS [8]. Under normal conditions, the immune function is in a state of dynamic equilibrium, and immune suppression and immune activation restrict each other [9]. However, the TIM of tumor tends to be immunosuppressive, thereby prompting tumor cells to evade immune surveillance [10]. How to relieve the immunosuppressive state of tumors is the focus and difficulty of tumor immunotherapy. The immune process of tumors is regulated by a variety of immune cells, and the killing of tumors by CD8^+^ T cells is the core of the whole process [11]. Therefore, elucidating the functional mechanism of CD8+ T cells is the key to the success of immunotherapy.

In recent years, a variety of immunotherapy drugs, such as PD-L1/PD-1 monoclonal antibody and CTLA4 monoclonal antibody, have been used in the treatment of malignant tumors and achieved satisfactory therapeutic effects, including OS [12]. However, in the application of immunotherapy, only a minority of patients benefit from immunotherapy [13]. It is currently believed that the cause of this dilemma is due to the abnormal immune microenvironment and immune cell function [14]. Therefore, developing a model that can accurately predict the efficacy of immunotherapy is crucial for patients of OS.

In this study, we obtained CD8^+^ T cell clusters and differentially expressed genes (DEGs). Next, we constructed a prognostic model based on the DEGs of CD8^+^, which was proved to have excellent predictive performance for the prognosis of OS patients. Moreover, we further revealed that the risk score of this model is closely related to the immune microenvironment of OS and multiple immune checkpoints, which can be used to predict immunotherapy response.

## 2. Materials and Methods

### 2.1. Acquisition of OS Single-Cell Sequencing Data and Transcriptome Data

Single-cell osteosarcoma data, including 11 OS patients, were obtained from the GEO dataset (https://www.ncbi.nlm.nih.gov/geo/, GSE152048). The OS transcriptome data TARGET-OS was downloaded from XENA (http://xenabrowser.net), including 88 OS samples.

### 2.2. Single-Cell RNA-Seq Data Quality Control and Data Processing

Single-cell samples of OS were processed by the R package Seurat package. Three-dimensional controls were applied to the original matrix of each cell: nFeature_RNA > 200 and percent.mt < 10 and nCount_RNA > 3. 3000 highly variable genes were identified using the FindVariableFeatures function, and principal component analysis- (PCA-) based dimensionality reduction was performed using RunPCA. Batch effects were removed on a sample-by-sample basis by the Harmony package. The distribution of cell components is mapped with R package “UMAP” with resolution = 0.5. Immune cells are distinguished from nonimmune cells based on the expression level of PRPDC (CD45). The resolution of cluster analysis of immune cells was 0.9. Findmarkers were used to screen signature genes, log2FC > 1 and *p* < 0.05.

### 2.3. Construction of Random Forest Prognostic Model

The characteristic genes we screened were firstly subjected to univariate prognostic analysis in TARGET-OS, and 6 genes were screened for inclusion in the prognostic model. Next, in this study, we randomly defined 70% of the TARGET-OS cohort as the training cohort and 30% as the validation cohort. The random forest prognostic model generates 1000 binary survival trees by default. When the number of survival trees increases to a certain number, the error rate curve tends to be stable.

### 2.4. Evaluation of the Predictive Power of the Model

We conducted the timeROC package to draw ROC curves to evaluate the predictive ability of the model. Next, patients were divided into high- and low-risk cohorts according to the model score best cutoff value, and the KM survival curve was used to compare the prognostic differences between the high-risk group and the low-risk group. Finally, univariate prognostic analysis was performed with model scores and clinical characteristics, and ROC curves were drawn.

### 2.5. GO and KEGG Enrichment Analysis

In this study, we carried out the GO analysis and KEGG to explore the value of our model risk score. The Database for Annotation, Visualization, and Integrated Discovery was used to integrate functional genomic annotations.

### 2.6. Evaluation to Immunotherapy Reactions

The ClusterProfiler package was used to perform GO and KEGG enrichment analysis of high- and low-risk patients [15]. OS immune cell infiltration analysis was performed by the ESTIMATE and XCELL algorithms [16]. The TIDE algorithm was used to evaluate the immune evasion ability of OS patients and predict the sensitivity to immunotherapy [17].

### 2.7. Statistics

In this study, R software (4.2.2) was used for calculation and statistical analysis of all data. We applied univariate and multivariate Cox regression analyses to assess the association of each factor with overall survival. The Student's *t*-test was conducted to compare the mean of different dataset. *p* < 0.05 were considered statistically significant.

## 3. Results

### 3.1. Identification of Immune Cells in OS Tissue

**Figure 1:**
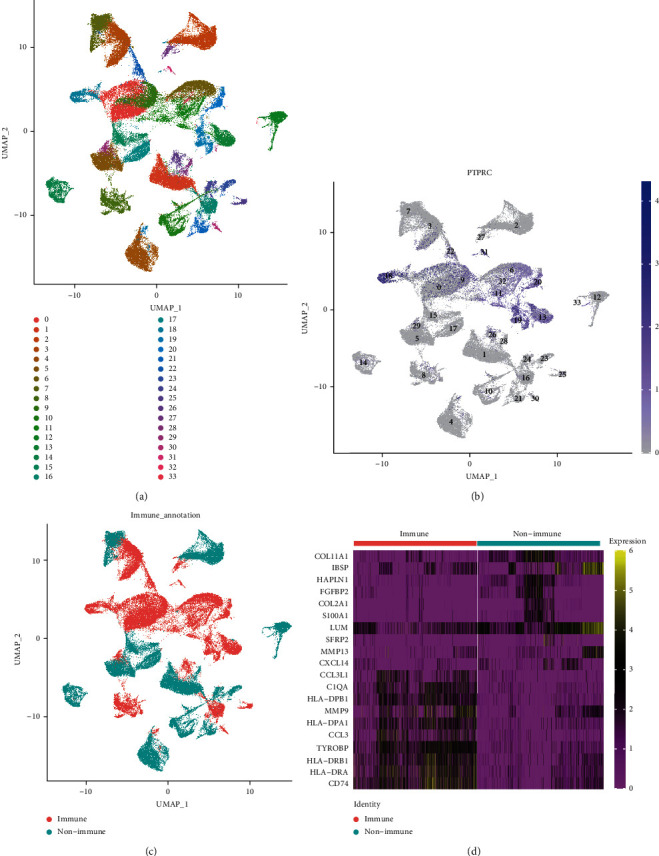
Identification of cell types in OS scRNA-seq sample. (a) UMAP plot of OS scRNA-seq data with 34 clusters (resolution = 0.5). (b) Expression of immune marker PTPRC (CD45) across all clusters, shown by UMAP plot. (c) Clustering of OS tissue via immune markers to obtain immune and nonimmune cells. (d) Heatmap of marker gene expression levels in different clusters.

To reveal the cellular heterogeneity of OS tissue, we collected single-cell RNA-sequencing (RNA-seq) datasets from 11 OS patients from the GEO database. We eliminated low-quality cells and identified 34 cell clusters from OS tissue by the dimensional reduction and clustering analysis (Figure 1(a)). PRPDC (CD45) is widely used as a characteristic marker of immune cells [18]. To clearly identify immune cells and nonimmune cells in OS tissue, we applied PRPDC to label immune cell clusters, and the results are shown in Figures 1(b) and 1(c). Finally, we further analyzed the signature genes of immune cell clusters and nonimmune cell clusters and found that immune cells highly expressed immune signature genes, which suggested that immune cells are well characterized by PTPRC (Figure 1(d)).

### 3.2. Definition of CD8^+^ T Cell Clusters and Extraction of Differentially Expressed Genes (DEGs)

**Figure 2:**
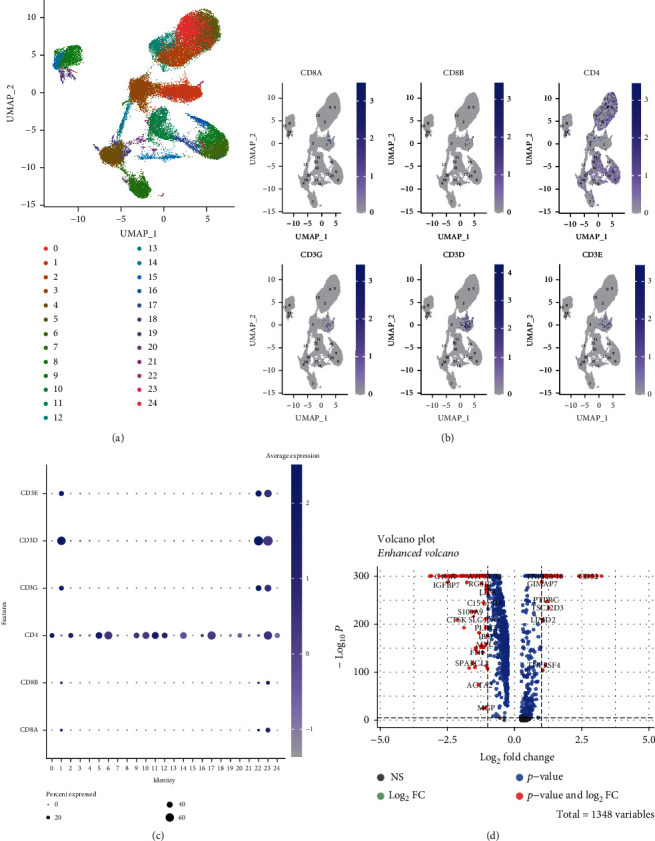
Identification of CD8^+^ T cell clusters and extraction of differentially expressed genes (DEGs). (a) UMAP plot of leukocyte scRNA-seq data with 25 clusters. (b) UMAP plots of single-cell expression levels of different marker genes. (c) Expression of different marker genes in immune cell subsets. (d) Analysis of DEGs between the 1st subset of immune cells and other cells.

We previously labeled OS tissues with corresponding marker genes and obtained immune cell clusters. We performed cluster analysis on the previous immune cell clusters and finally obtained 25 cell clusters (Figure 2(a)). Next, we applied CD8A and CD8B to label CD8^+^ T cell clusters, CD4 to label CD4^+^ T cell clusters, and CD3G, CD3D, and CD3E to label T cell clusters, respectively. The results indicated that the cluster analysis had good clustering performance (Figure 2(b)). In addition, to further isolate CD8^+^ T cells, we analyzed the expression levels of marker genes in different immune cell clusters. The results suggested that cluster1 could represent the optimal choice for CD8^+^ T cell clusters (Figure 2(c)). We performed differentially expressed gene (DEG) analysis on the obtained CD8^+^ cell clusters and the remaining cells (Log_2_FC > 1, *p* < 0.05) and finally obtained 59 genes with statistical significance (Figure 2(d) and Supplementary Table 1).

### 3.3. Construction of Risk Score Model Based on DEGs in CD8^+^ T Cells (CD8-DEGs)

**Figure 3:**
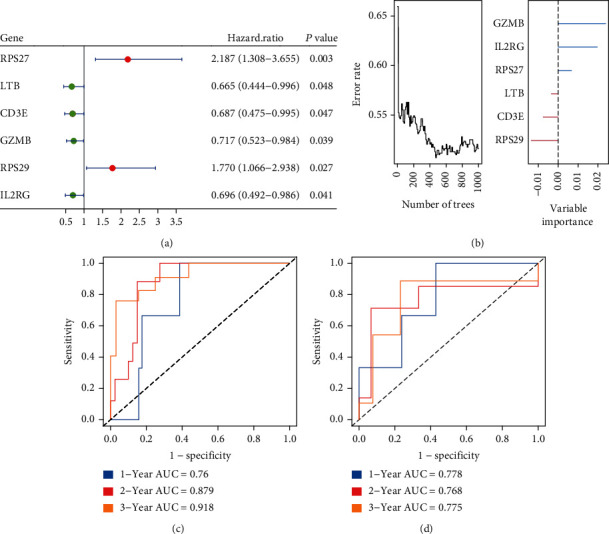
Construction of risk models. (a) Univariate regression analysis of CD8-DEGs. (b) Plot of the out of bag (OOB) prediction error rate for each tree constructed in the CD8-DEG model (left panel). Plot of variable importance in the CD8-DEG model (right panel). (c, d) ROC curves showed the predictive efficiency of the CD8-DEG risk score for 1-, 3-, and 5-year survival in the training and validation cohorts.

Given the DEG analysis by CD8^+^ T cells, we finally obtained 59 genes. We performed univariate analysis in the TARGET-OS dataset and finally obtained 6 genes (RPS27, LTB, CD3E, GZMB, RPS29, and IL2RG). The results showed that RPS27 and RPS29 were high-risk factors, while LTB, CD3E, GZMB, and IL2RG were low-risk factors in OS (Figure 3(a)). In addition, we divided the TARGET-OS patients into training cohort and validation cohort by 70% and 30%. We adopted these 6 genes and constructed a random survival forest model (CD8-DEGs model) based on the DEGs of CD8^+^ T cells. As shown in Figure 3(b), our model exhibited high accuracy, and GZMB and IL2RG play a major role in this model. We further evaluated the accuracy of our model by ROC curve, and the results demonstrated that in the training cohort, the ROC curve area reached 0.75, 0.879, and 0.918 at 1, 3, and 5 years (Figure 3(c)). Likewise, in the validation cohort, the ROC curve areas reached 0.778, 0.768, and 0.775 at 1, 3, and 5 years (Figure 3(d)).

### 3.4. Evaluating the Performance of the CD8-DEG Model

**Figure 4:**
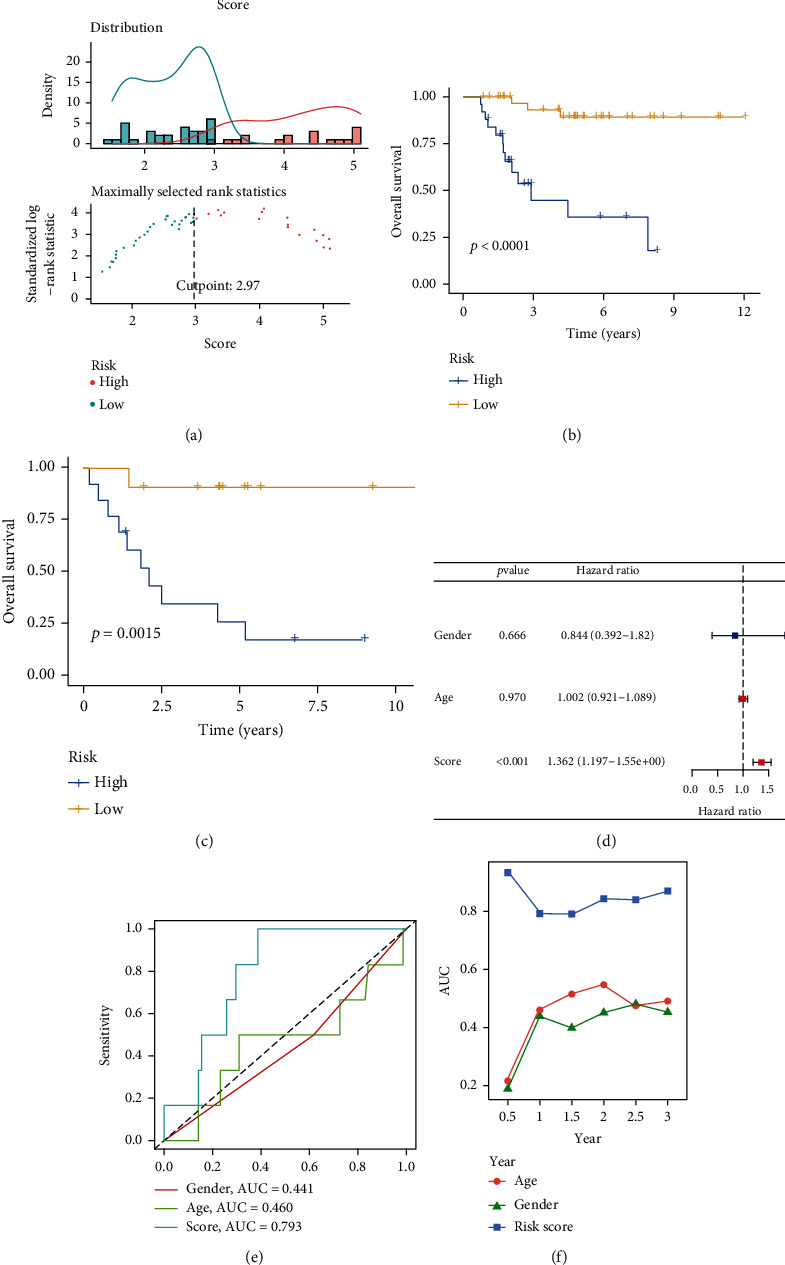
Evaluation of risk models for CD8-DEGs. (a) OS patients were divided into high- and low-risk groups according to the optimal cutoff (2.97) value. (b, c) Comparison of OS between high- and low-risk scores in the training and validation cohorts. (d) Univariate analysis of CD8-DEG risk scores and clinical characteristics. (e, f) Comparison of the predictive power of CD8-DEG risk models and their clinical characteristics.

To further investigate the value of CD8-DEGs model, we assessed the predictive power of this model by various measures. As shown in Figure 4(a), we took 2.97 as the cutoff value to classify the TARGET-OS cohort patients into high- and low-risk cohorts. The results demonstrated that high-risk patients had worse prognosis in both the training and validation cohorts (Figures 4(b) and 4(c)). In addition, we further adopted the corresponding clinical characteristics (gender and age) and their risk scores for univariate analysis. We found that risk score was a high-risk factor for OS patient prognosis (=1.362) (Figure 4(d)). Meanwhile, the area under the ROC curve of the risk score reached 0.793 (Figure 4(e)). Finally, *C*-index analysis showed that risk scores had higher AUC curve values compared to gender and age (Figure 4(f)). These data strongly indicated that the CD8-DEG model has good performance in predicting the prognosis of OS patients.

### 3.5. Pathway Enrichment Analysis of Risk Scores for the CD8-DEG Model

**Figure 5:**
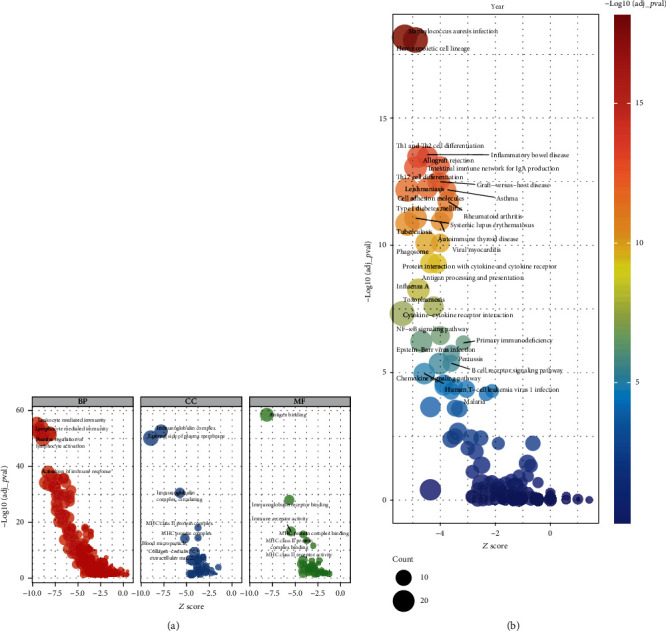
Pathway enrichment analysis of high- and low-risk scores in CD8-DEG model. (a) GO analysis of CD8-DEGs model. (b) KEGG analysis of CD8-DEG model.

The CD8-DEG model showed excellent ability in predicting the prognosis of OS patients. To initially revealed the mechanism, we performed GO and KEGG enrichment analysis. The results of GO analysis showed that the risk score of the CD8-DEGs model was closely related to multiple immune functions, such as leukocyte-mediated immunity, activation of immune response, MHC complexes, and antigen binding (Figure 5(a)). KEGG analysis revealed that the risk score of the CD8-DEG model was associated with multiple immune-related pathways, such as Th1 and Th2 cell differentiation, intestinal immune network for IgA production, cell adhesion molecules, cytokine-cytokine receptor interactions and NF-kappa B pathway (Figure 5(b)).

### 3.6. Correlations between Risk Scores and OS Immune Profiles

**Figure 6:**
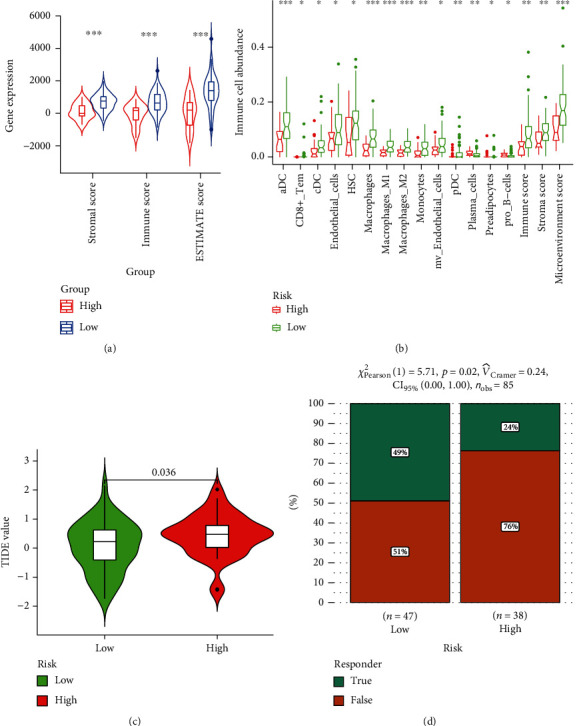
Correlation of risk scores with immune profiles of OS. (a) Comparison of the stromal score, immune score, and ESTIMATE score between high-risk and low-risk groups. (b) Comparison of immune cell infiltration between high-risk and low-risk groups. (c) Comparison of TIDE value between high-risk and low-risk groups. (d) Comparison of the response to immunotherapy in patients with OS in high-risk and low-risk groups.

The previous results indicated that the risk score of CD8-DEG model is closely related to multiple immune pathways. We analyzed the association of risk scores and OS immune profile. As shown in Figure 6(a), low-risk patients had higher scores in stromal score, immune score, and ESTIMATE score. We applied CIBERSORT to assess the effect of risk score on immune cell infiltration. It was found that high-risk patients negatively regulate the infiltration of various immune cells, such as CD8^+^ T cells, macrophages, and M1-like macrophages (Figure 6(b)). In recent years, TIDE has been widely used as an indicator for tumor immune evasion ability. As presented in Figure 6(c), high-risk patients had lower TIDE scores. Finally, we further assessed whether risk scores could be used as a predictor of response to immunotherapy in OS. The results showed that OS patients with high-risk scores tended to be insensitive to immunotherapy (Figure 6(d)).

### 3.7. Correlation of Risk Scores with Immune Checkpoints and Validation of Risk Genes

**Figure 7:**
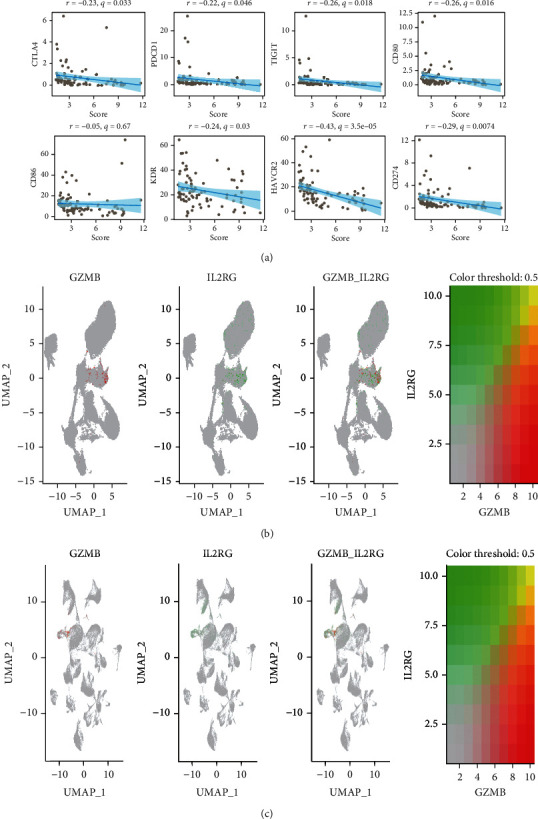
Correlation of risk scores with immune checkpoints and validation of risk genes. (a) Correlation analysis of immune checkpoints and risk scores. (b) Expression of GZMB and IL2RG in UMAP plots of OS scRNA-seq data. (c) Expression of GZMB and IL2RG in UMAP plots of leukocyte scRNA-seq data.

Due to the immune checkpoints play an important role in regulating immune cell function, we further analyzed the relationship between risk scores and immune checkpoints. As shown in Figure 7(a), the risk score was negatively correlated with the expression of multiple immune checkpoints, including CTLA4, PDCD1, TIGIT, CD80, CD86, KDR, HAVCR2, and CD274. Finally, we further verified the expression of GZMB and IL2RG in OS scRNA-seq and leukocyte scRNA-seq data. The results revealed that GZMB and IL2RG similarly clustered in the UMAP plot of OS scRNA-seq data (Figure 7(b)). Similarly, GZMB and IL2RG were clustered in UMAP plots of leukocyte scRNA-seq data (Figure 7(c)).

## 4. Discussion

The poor prognosis of OS has plagued and threatens the physical and mental health of human beings. Immunotherapy is considered a promising treatment for improving OS prognosis [19]. Given the current dilemma of immunotherapy for OS, it is urgent to develop an effective method to predict the response to immunotherapy. In this study, we first downloaded and processed the single-cell sequencing data of OS from the GEO database. Next, we clustered the above data and further marked it by specific markers to obtain immune cell clusters and nonimmune cells. In addition, we performed cluster analysis on the obtained immune cell clusters, while applying specific markers to extract CD8^+^ T cell clusters, and obtained DEGs of CD8^+^ T cells by gene differential analysis. Furthermore, we combined TCGA-OS cohort data to perform univariate regression analysis on DEGs to obtain prognostic-related genes. A random forest model (CD8-DEG model) was constructed for the above-mentioned prognosis-related genes, and further verification found that it has good predictive performance. Moreover, we revealed that the risk score of CD8-DEG model was significantly associated with the immune profile and could also be used as a predictor of OS immunotherapy response. Finally, we demonstrated the correlation between risk score and the expression of multiple immune checkpoints.

Prognostic models based on various functional gene sets of tumors, such as ferroptosis, pyroptosis, and autophagy, have gradually become a research hotspot in recent years. Tang et al. constructed a ferroptosis-related lncRNA prognostic model in head and neck squamous cell carcinoma, and the area under the ROC curve of the model risk score reached 0.782 [20]. Zhang et al. analyzed the expression levels of pyroptosis-related genes in human endometrial cancer and constructed a prognostic model based on pyroptosis-related genes. The ROC value of the model was 0.613 [21]. Duan et al. analyzed the expression levels of autophagy-related gene lncRNAs in colorectal cancer, obtained 11 lncRNAs related to autophagy, and further constructed a prognostic model. The ROC area of this model reached 0.808 [22]. In this study, constructed a prognostic model based on the differentially expressed genes of CD8^+^ cells, the area under the ROC curve was 0.793. The prognostic model of DEGs based on CD8^+^ T cells we established has a predictive ability that is not inferior to other previous prognostic models of functional gene sets, which provided a valuable reference for evaluating the prognosis of OS.

Single-cell transcriptome sequencing is performed by analyzing the mRNA expression level of each cell in a sample. In this study, we performed two cluster analyses. We first performed cluster analysis on the single-cell data of OS, resulting in 34 cell clusters. Then, we defined immune cell clusters by PTPRC. Next, we again performed cluster analysis on the immune cell clusters. In this study, we applied two clustering analyses to more precisely define CD8^+^T cells. This is more reliable than previous analysis of BULK sequencing data.

The model we established has a high application prospect, but there are also some obvious shortcomings. First, all data are derived from public data and lack in vivo and in vitro validation. Second, the specific mechanisms by which the model predicts immune signatures and immunotherapy have not been further explored. These are worthy of further clarification in our follow-up research.

In conclusion, the CD8-DEG model can not only be used to analyze the immune profile of OS but also can be used as a marker to evaluate the efficacy of OS on immunotherapy.

## Data Availability

All data generated or analyzed in this study are available from the corresponding author for reasonable request.

## Supplementary Materials

Supplementary Materials Supplementary Table: differentially expressed genes between CD8^+^ cell clusters and remaining cells.
