# Supplementary material for: The Prognostic Model Established by the Differential Expression Genes Based on CD8+ T Cells to Evaluate the Prognosis and the Response to Immunotherapy in Osteosarcoma
Source: Mediators Inflamm. 2023 Feb 9;2023:6563609. doi: 10.1155/2023/6563609 (PMC9934978; doi:10.1155/2023/6563609)
Supplement: Supplementary Materials — Supplementary Table: differentially expressed genes between CD8+ cell clusters and remaining cells. [file 6563609.f1.doc]

| id | p_val | avg_log2FC | pct.1 | pct.2 | p_val_adj |  |
| --- | --- | --- | --- | --- | --- | --- |
| TNFRSF18 | 0 | 1.169469797 | 0.135 | 0.013 | 0 |  |
| CD52 | 0 | 2.756554191 | 0.734 | 0.127 | 0 |  |
| LCK | 0 | 1.366596093 | 0.27 | 0.01 | 0 |  |
| CD2 | 0 | 2.450034042 | 0.518 | 0.023 | 0 |  |
| RPS27 | 0 | 1.158170438 | 0.994 | 0.979 | 0 |  |
| CD48 | 0 | 1.177261779 | 0.334 | 0.158 | 0 |  |
| CD247 | 0 | 1.307343849 | 0.236 | 0.009 | 0 |  |
| XCL2 | 0 | 1.698974883 | 0.155 | 0.005 | 0 |  |
| LINC01871 | 0 | 1.020075305 | 0.164 | 0.005 | 0 |  |
| GNLY | 0 | 2.900645713 | 0.158 | 0.011 | 0 |  |
| DUSP2 | 0 | 1.595870814 | 0.429 | 0.219 | 0 |  |
| CXCR4 | 0 | 1.597272756 | 0.554 | 0.282 | 0 |  |
| CYTIP | 0 | 1.103584677 | 0.287 | 0.12 | 0 |  |
| TUBA4A | 0 | 1.094739427 | 0.214 | 0.051 | 0 |  |
| TIGIT | 0 | 1.117981641 | 0.149 | 0.005 | 0 |  |
| HOPX | 0 | 1.070208018 | 0.178 | 0.026 | 0 |  |
| IL7R | 0 | 1.640796537 | 0.294 | 0.069 | 0 |  |
| GZMK | 0 | 2.396618455 | 0.316 | 0.015 | 0 |  |
| GZMA | 0 | 2.814796262 | 0.46 | 0.024 | 0 |  |
| LTB | 0 | 2.907415154 | 0.526 | 0.053 | 0 |  |
| STK17A | 0 | 1.235506037 | 0.289 | 0.103 | 0 |  |
| IL2RG | 0 | 1.336810529 | 0.294 | 0.1 | 0 |  |
| ITM2A | 0 | 1.215947452 | 0.287 | 0.071 | 0 |  |
| RARRES3 | 0 | 1.285745782 | 0.335 | 0.107 | 0 |  |
| CTSW | 0 | 1.446411704 | 0.192 | 0.008 | 0 |  |
| TBC1D10C | 0 | 1.147227366 | 0.232 | 0.034 | 0 |  |
| CD3E | 0 | 1.545783303 | 0.314 | 0.011 | 0 |  |
| CD3D | 0 | 2.713405199 | 0.601 | 0.025 | 0 |  |
| CD3G | 0 | 1.556408286 | 0.296 | 0.009 | 0 |  |
| SPOCK2 | 0 | 1.05945515 | 0.192 | 0.009 | 0 |  |
| CD27 | 0 | 1.288379563 | 0.212 | 0.007 | 0 |  |
| KLRB1 | 0 | 2.870555943 | 0.376 | 0.015 | 0 |  |
| CD69 | 0 | 2.916738951 | 0.601 | 0.082 | 0 |  |
| IFNG | 0 | 1.538151357 | 0.168 | 0.007 | 0 |  |
| BTG1 | 0 | 1.367402693 | 0.778 | 0.581 | 0 |  |
| GZMH | 0 | 1.713938877 | 0.195 | 0.006 | 0 |  |
| GZMB | 0 | 1.320348442 | 0.118 | 0.01 | 0 |  |
| RPS29 | 0 | 1.123383567 | 0.971 | 0.938 | 0 |  |
| EVL | 0 | 1.122494135 | 0.328 | 0.14 | 0 |  |
| ISG20 | 0 | 1.417902142 | 0.311 | 0.067 | 0 |  |
| IL32 | 0 | 3.065612496 | 0.715 | 0.141 | 0 |  |
| CORO1A | 0 | 1.332137799 | 0.507 | 0.268 | 0 |  |
| ACAP1 | 0 | 1.354199948 | 0.297 | 0.038 | 0 |  |
| CCL5 | 0 | 3.231271128 | 0.556 | 0.123 | 0 |  |
| CD7 | 0 | 1.596051819 | 0.269 | 0.013 | 0 |  |
| CST7 | 0 | 1.744395191 | 0.307 | 0.031 | 0 |  |
| GZMM | 0 | 1.448085684 | 0.256 | 0.008 | 0 |  |
| JUNB | 0 | 1.463851022 | 0.811 | 0.659 | 0 |  |
| DNAJB1 | 0 | 1.336494613 | 0.739 | 0.591 | 0 |  |
| HCST | 0 | 1.321168603 | 0.563 | 0.312 | 0 |  |
| NKG7 | 0 | 2.616670887 | 0.338 | 0.042 | 0 |  |
| APOBEC3G | 0 | 1.21080242 | 0.241 | 0.073 | 0 |  |
| GIMAP7 | 5.12E-293 | 1.011000622 | 0.332 | 0.159 | 1.30E-288 |  |
| PTPRC | 5.56E-253 | 1.200929924 | 0.457 | 0.323 | 1.42E-248 |  |
| TSC22D3 | 4.54E-240 | 1.26308675 | 0.462 | 0.33 | 1.16E-235 |  |
| LIMD2 | 1.29E-214 | 1.033325091 | 0.319 | 0.182 | 3.28E-210 |  |
| STK17B | 1.81E-212 | 1.044332697 | 0.282 | 0.148 | 4.60E-208 |  |
| TNFRSF4 | 6.05E-120 | 1.13598816 | 0.126 | 0.052 | 1.54E-115 |  |
| JUND | 4.68E-109 | 1.043425328 | 0.496 | 0.431 | 1.19E-104 |  |
